# Parkinson's disease diffusion MRI is not affected by acute antiparkinsonian medication

**DOI:** 10.1016/j.nicl.2017.02.012

**Published:** 2017-02-16

**Authors:** Jae Woo Chung, Roxana G. Burciu, Edward Ofori, Priyank Shukla, Michael S. Okun, Christopher W. Hess, David E. Vaillancourt

**Affiliations:** a Department of Applied Physiology and Kinesiology, University of Florida, Gainesville, FL, USA; b Department of Neurology and Center for Movement Disorders and Neurorestoration, University of Florida, Gainesville, FL, USA; c Department of Biomedical Engineering, University of Florida, Gainesville, FL, USA

**Keywords:** dMRI, Parkinson's disease, Antiparkinsonian medication, Free-water, Free-water corrected fractional anisotropy

## Abstract

**Objective:**

A prior longitudinal study demonstrates that free-water diffusion magnetic resonance imaging (dMRI) tracks progression in the substantia nigra (Ofori et al., 2015b). Here, we test the acute effects of antiparkinsonian medication on this established imaging progression marker for the first time.

**Methods:**

Fifteen PD patients underwent dMRI OFF and ON-medication one day apart. ON-medication, patients were tested approximately 45 min after their usual dose of antiparkinsonian medication. OFF-medication, patients were tested after not taking antiparkinsonian medication for > 12 h. OFF and ON-medication was counter-balanced across subjects. For dMRI, we computed free-water and free-water corrected fractional anisotropy (FA_t_) within the following regions: caudate, putamen, substantia nigra, and subthalamic nucleus.

**Results:**

ON-medication significantly reduced parkinsonian motor symptoms compared with OFF-medication (*p* < 0.001). dMRI measures (free-water and FA_t_) were not different between the OFF and ON-medication conditions.

**Conclusions:**

Administration of an acute does of anti-parkinsonian medication in PD does not affect free-water and FA_t_ in key nigrostriatal structures. Free-water and FA_t_ biomarkers reflect the chronic state of the nigrostriatal circuit, and therefore are potential viable biomarkers for disease-modifying therapeutic studies in PD.

## Highlights

• Effects of dopaminergic medication on PD progression markers
• Investigate dMRI biomarkers in early PD
• Acute PD medication does not alter the progression markers.

## 1 Introduction

Parkinson's disease (PD) is the second most common age-related neurodegenerative disorder and is characterized by the presence of tremor, rigidity, bradykinesia, and impaired posture and gait. Histologically, PD results in a significant reduction of dopaminergic cells in the substantia nigra pars compacta (SNc) (Fearnley and Lees, 1991, Stocchi and Olanow, 2003) and a dopamine deficiency in specific nuclei of the basal ganglia. Along with this substantial neuronal loss, the main pathological hallmark of PD is the presence of intracellular α-synuclein-positive inclusions, known as Lewy bodies and Lewy neurites, throughout subcortical and cortical brain regions (Braak et al., 2003, Stocchi and Olanow, 2003). Developing non-invasive methods to assay features reflecting this degeneration is a critical area of ongoing research.

Numerous studies using diffusion magnetic resonance imaging (dMRI) have detected altered diffusion in the substantia nigra of PD compared with control individuals (Cochrane and Ebmeier, 2013, Du et al., 2012, Langley et al., 2016, Ofori et al., 2015a). Using free-water analysis of dMRI signals revealed that free-water in the substantia nigra increased longitudinally over one year in PD with no changes in a control group (Ofori et al., 2015b). This dMRI progression biomarker of the substantia nigra holds promise for being useful in clinical trials testing disease-modifying therapies for PD.

A key unanswered question is whether free-water in the substantia nigra is influenced by antiparkinsonian medication. This is important because most disease modifying clinical trials will include PD patients on levodopa and it is necessary to determine if administration of levodopa alters this biomarker of progression. If administering antiparkinsonian medication affects dMRI measurements, then they likely would represent faster changes in the basal ganglia than would be useful for a biomarker of disease progression, whereas if they are not influenced by acute antiparkinsonian medication then they may be well suited as progression biomarkers that would reflect the chronic state of basal ganglia microstructure. Here, we evaluated for the first time whether administration of antiparkinsonian medication was associated with changes in diffusion MRI free-water and free-water corrected fractional anisotropy in the substantia nigra and other basal ganglia structures.

## 2 Materials and methods

### 2.1 Participants

A total of 15 PD patients were diagnosed by a movement disorder specialist using established criteria (Hughes et al., 2001), and recruited from University of Florida Center for Movement Disorders and Neurorestoration. The study was approved by the Institutional Review Board and all participants provided informed consent prior to participating in the study (Table 1).

**Table 1:** Demographics and clinical data.

Demographics | Clinical Data	OFF-medication	ON-medication	*p*-Value	*pFDR*
Sample size	15			
Age, yrs	62.00 (10.51)			
Gender (M | F)	9 | 6			
Disease duration, yrs	5.17 (4.78)			
Hoehn and Yahr stage (OFF-med)	1.67 (0.62)			
Total LEDD	674.00 (364.59)			
MDS-UPDRS-III – Total	16.80 (8.51)	13.13 (6.98)	< 0.001	< 0.001
MoCA	27.20 (2.18)	27.67 (1.54)	0.301	0.597

Data are count or mean (± SD) (*n* = 15). Disease duration is defined as time since diagnosis. *p*-Values are uncorrected. *pFDR* values are FDR corrected. Abbreviations: F = females; LEDD = levodopa equivalent daily dose; M = males; MoCA = Montreal Cognitive Assessment; MDS-UPDRS-III = the motor section of the Movement Disorder Society Unified Parkinson's Disease Rating Scale.

### 2.2 Procedures

The experiments for the patients were performed on two consecutive days; (i) OFF-medication; and (ii) ON-medication. The order of testing day was counter-balanced across subjects. PD patients were tested in the practically defined off state (Langston et al., 1992), with testing following a 12-hour withdrawal from anti-parkinsonian medication. Testing ON-medication occurred approximately 45 min corresponding to the time required for levodopa plasma level to reach its peak (Brooks, 2008) after taking the patient-specific dose of antiparkinsonian medication (Table 2). Each patient underwent dMRI evaluation, and assessment of motor symptoms and cognitive status on two consecutive days. Motor symptoms and cognitive status were assessed using part III of the Movement Disorder Society Unified Parkinson's Disease Rating Scale (MDS-UPDRS-III) and the Montreal Cognitive Assessment (MoCA), respectively (Goetz et al., 2008, Nasreddine et al., 2005). The total levodopa equivalent daily dose (LEDD) and the single dose LEDD before dMRI scan were calculated according to previously published recommendations (Tomlinson et al., 2010) (Table 1).

**Table 2:** Patient antiparkinsonian medications.

Subject	Medication	Total LEDD (mg)/a day	Single dose LEDD (mg)	
			LEDD (mg)	% of Total LEDD
PD 1	Carbidopa/Levodopa (Atamet, Sinemet) Pramipexole	1100	300	27.27
PD 2	Carbidopa/Levodopa (Atamet, Sinemet) Amantadine (Symmetrel)	1450	350	24.14
PD 3	Carbidopa/Levodopa (Atamet, Sinemet) Pramipexole ER (Mirapex)	1050	650	61.90
PD 4	Rotigotine Transdermal Patch (Neupro) Carbidopa/Levodopa/Entacapone (Stalevo)	1260	396	31.43
PD 5	Carbidopa/Levodopa (Atamet, Sinemet) Rasagiline (Azilect)	500	200	40.00
PD 6	Carbidopa/Levodopa (Atamet, Sinemet)	600	300	50.00
	Rasagiline (Azilect)			
	Pramipexole			
PD 7	Carbidopa/Levodopa (Atamet, Sinemet) Rasagiline (Azilect)	500	200	40.00
PD 8	Carbidopa/Levodopa (Atamet, Sinemet) Selegiline (Eldepryl)	500	150	30.00
PD 9	Carbidopa/Levodopa (Atamet, Sinemet) Rasagiline (Azilect)	400	200	50.00
PD 10	Carbidopa/Levodopa (Atamet, Sinemet) Rasagiline (Azilect)	500	200	40.00
PD 11	Carbidopa/Levodopa (Atamet, Sinemet)	500	100	20.00
PD 12	Carbidopa/Levodopa Sustained Release (Sinemet CR)	300	150	50.00
PD 13	Carbidopa/Levodopa Sustained Release (Sinemet CR) Selegiline (Eldepryl)	750	150	20.00
PD 14	Carbidopa/Levodopa (Atamet, Sinemet) Rasagiline (Azilect)	400	200	50.00
PD 15	Carbidopa/Levodopa (Atamet, Sinemet)	300	100	33.33

Abbreviations: LEDD = total levodopa equivalent daily dose; PD = Parkinson's disease.

### 2.3 MRI data acquisition protocol

MRI was performed on a 3T system (Philips Achieva) equipped with a 32-channel quadrature volume head coil. Head movement was minimized by foam padding within the coil and scanner noise was attenuated using a combination of earplugs and circumaural headphones. Whole brain diffusion imaging data was acquired using a single-shot spin echo EPI sequence: repetition time = 7748 ms, echo time = 86 ms, flip angle = 90°, field of view = 224 × 224 mm, voxel size = 2 mm isotropic with no gap between slices (*n* = 60), diffusion gradient (monopolar) directions = 64, diffusion gradient timing DELTA/delta = 42.4/10 ms, *b*-values: 0, 1000 s/mm^2^, fat suppression using SPIR, in-plane, SENSE factor = 2.

### 2.4 Diffusion MRI data analysis

FMRIB Software Library (FSL, http://www.fmrib.ox.ac.uk/fsl/) and custom UNIX shell scripts were used to preprocess the data (Ofori et al., 2015b). Diffusion MRI data were not flipped. Each diffusion scan was eddy current and head motion corrected. Diffusion gradients were compensated for rotations, and non-brain tissue was removed. Free-water and free-water-corrected fractional anisotropy (FA_t_) maps were calculated from the corrected volumes using a custom code written in MATLAB R2013a (The Mathworks, Natick, MA) (Pasternak et al., 2009). To create the free-water map, a minimization procedure was used that fit a bi-tensor model to each voxel to quantify its fractional volume of free-water. The free-water component was then eliminated from each voxel to generate the FA_t_ maps. The *b*0 images were normalized to a MNI-T_2_ template (2 × 2 × 2 mm) by an affine transformation with 12 degrees of freedom and trilinear interpolation using FLIRT (http://fsl.fmrib.ox.ac.uk/fsl/fslwiki/FLIRT). The spatial transformation parameters obtained from this step were applied to the free-water and FA_t_ maps. Since FLIRT-transformed images are not sufficient for accurate registration, we used regions of interest hand-drawn on the *b*0 image of each subject in MNI space. The regions were drawn by an experienced rater who was blinded to the free-water image and medication state, and then used to extract values from the corresponding free-water and FA_t_ maps. The size of each region of interest was chosen to fit within the brain structure across all subjects. Bilateral regions of interest (ROI) were drawn in the following areas (number of voxels per hemisphere, *n*): anterior substantia nigra (*n* = 8), posterior substantia nigra (*n* = 8), putamen (*n* = 88), caudate nucleus (*n* = 68), and subthalamic nucleus (*n* = 8) (Burciu et al., 2016, Ofori et al., 2015b, Planetta et al., 2015, Prodoehl et al., 2013).

### 2.5 Statistical analysis

Statistical analyses were performed in SPSS (version 24.0; IBM Corp). To examine the effect of antiparkinsonian medication, we compared each dependent variable OFF-medication with the ON-medication dependent variable using two-tailed paired *t*-test. The significance level was set at *p* < 0.05. All *p*-values reported in the Results section of the manuscript were corrected for multiple comparisons using the Benjamini-Hochberg false discovery rate (FDR) method (Benjamini and Hochberg, 1995). Uncorrected *p*-values are also reported. Power analysis was performed in G*Power (version 3.1.9.2) (Faul et al., 2007). We report required sample size at 80% power for future studies that would attempt to detect an effect of acute antiparkinsonian medication on free-water and FA_t_ measures in the five ROIs (Table 4). The required sample size was calculated based on mean differences in free-water and FA_t_ between the OFF and ON-medication conditions at three levels of α error (0.05, 0.01, and 0.005).

## 3 Results

### 3.1 Clinical and force data

Table 1 shows the demographic and clinical results. ON-medication significantly reduced parkinsonian motor symptoms compared with OFF-medication (*pFDR* < 0.001). There was no effect of medication for the MoCA.

### 3.2 Diffusion MRI data

**Fig. 1:**
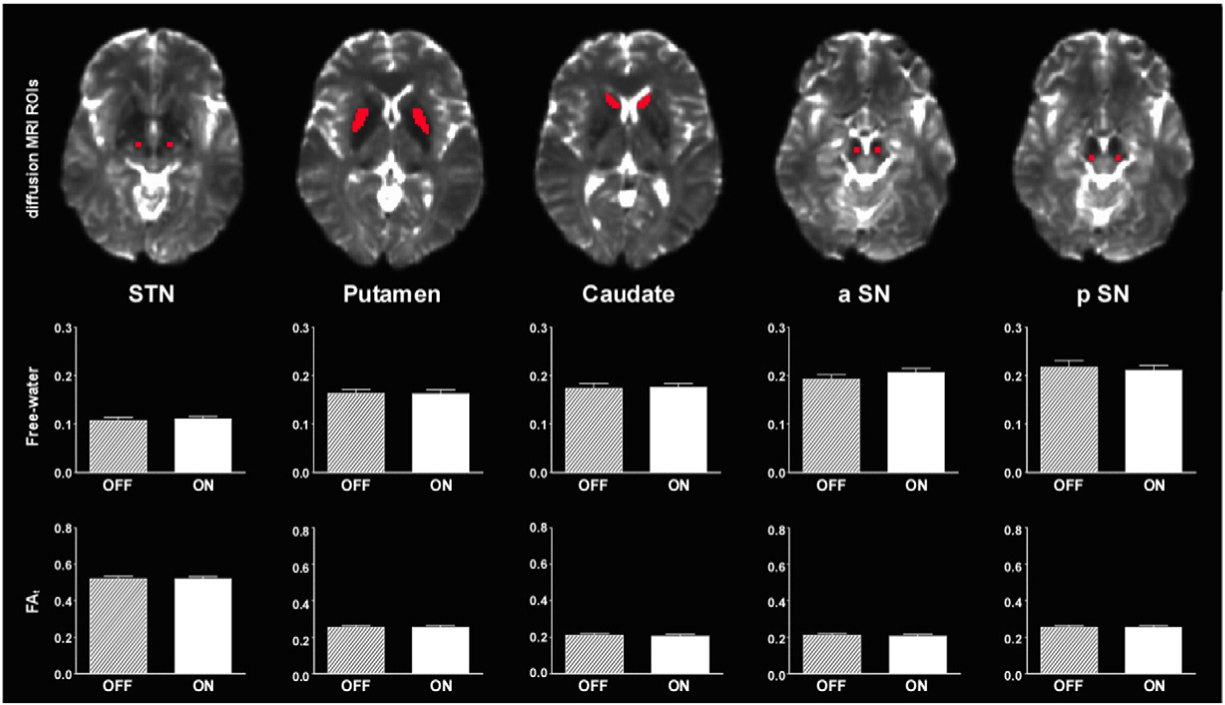
dMRI measurements (Free-water and FA_t_) differences between OFF and ON-medication. Mean (± SE) dMRI measurements in the 5 dMRI-ROIs respectively. Abbreviation: a = anterior; FA_t_ = free-water corrected fractional anisotropy; p = posterior; SN = substantia nigra; STN = subthalamic nucleus.

Fig. 1 and Table 3 show the results of free-water and FA_t_ in the 5 ROIs of diffusion MRI. The results of free-water showed no significant effect of antiparkinsonian medication in the caudate, putamen, subthalamic nucleus, anterior substantia nigra, and posterior substantia nigra. Also, the results of FA_t_ showed no significant effect of antiparkinsonian medication in the caudate, putamen, subthalamic nucleus, anterior substantia nigra, and posterior substantia nigra (Table 3). The uncorrected *p*-values did not approach significance.

**Table 3:** Diffusion MRI.

Diffusion MRI (FW | FAt)	OFF-medication	ON-medication	*p*-Value	*pFDR*
FW - Caudate	0.176 (0.038)	0.179 (0.032)	0.569	0.951
FW - Putamen	0.164 (0.035)	0.162 (0.033)	0.358	0.951
FW - STN	0.106 (0.024)	0.108 (0.019)	0.739	0.951
FW - SN anterior	0.191 (0.038)	0.201 (0.032)	0.274	0.951
FW - SN posterior	0.229 (0.070)	0.221 (0.054)	0.390	0.951
FAt - Caudate	0.209 (0.038)	0.210 (0.036)	0.961	0.972
FAt - Putamen	0.246 (0.036)	0.246 (0.033)	0.972	0.972
FAt - STN	0.526 (0.039)	0.524 (0.022)	0.761	0.951
FAt - SN anterior	0.627(0.084)	0.631 (0.068)	0.716	0.951
FAt - SN posterior	0.640 (0.056)	0.633 (0.063)	0.523	0.951

OFF and ON-medication mean (± SD) diffusion MRI evaluations (*n* = 15). *p*-Values are uncorrected. *pFDR* values are FDR corrected. Abbreviations: FAt = free-water corrected fractional anisotropy; FW = free-water; SN = substantia nigra; STN = subthalamic nucleus.

The results of the power analysis based on mean differences in free-water and FA_t_ between the OFF and ON-medication conditions are listed in Table 4. To detect a significant effect of antiparkinsonian medication on free-water in the posterior substantia nigra at 80% power and α error = 0.005, the required sample size would be 258 patients. For the same α error = 0.005, 470 patients would be needed to detect significant effects of antiparkinsonian medication on FA_t_ in the posterior substantia nigra. These power analyses assume the same mean difference and standard deviation as found in the current study.

**Table 4:** Sample size estimation.

Diffusion MRI (FW | FAt)	Δ Mean (OFF-ON)	Δ SD (OFF-ON)	Effect size	Sample size (Power 80%)		
				0.05	0.01	0.005
FW - Caudate	− 0.0027	0.0177	− 0.1503	350	521	594
FW - Putamen	0.0018	0.0075	0.2457	132	197	225
FW - STN	− 0.0018	0.0210	− 0.0878	1,021	1,519	1,732
FW - SN anterior	− 0.0090	0.0307	− 0.2940	93	139	158
FW - SN posterior	0.0077	0.0337	0.2292	152	226	258
FAt - Caudate	− 0.0002	0.0148	− 0.0128	47,690	70,963	80,890
FAt - Putamen	− 0.0001	0.0121	− 0.0091	94,974	141,319	161,088
FAt - STN	0.0024	0.0295	0.0799	1,230	1,831	2,087
FAt - SN anterior	− 0.0037	0.0387	− 0.0958	857	1,275	1,454
FAt - SN posterior	0.0068	0.0402	0.1691	277	412	470

Power analysis for diffusion MRI measurements (FW and FAt) (*n* = 15). OFF and ON-medication paired differences Mean and SD. Sample size is calculated by 80% power at each α error (0.05, 0.01, and 0.005). Abbreviations: FAt = free-water corrected fractional anisotropy; FW = free-water; SN = substantia nigra; STN = subthalamic nucleus; Δ = differences.

## 4 Discussion

The current study examined if an acute administration of dopaminergic medication affects free-water and FA_t_ in the basal ganglia. Although dopaminergic medication reduced the MDS-UPDRS III score, there was no evidence that taking dopaminergic medication affected the dMRI regional measures previously shown to progress with PD. These findings are discussed in the context of the recent literature advancing these measures as potential biomarkers of progression in PD.

Prior studies using dMRI have shown significant differences in either fractional anisotropy (FA) or mean diffusivity in the substantia nigra in PD compared to healthy controls (Du et al., 2012, Langley et al., 2016, Schwarz et al., 2013). A Cochrane meta-analysis concluded that diffusion imaging abnormalities are common findings across multiple studies and suggests dMRI could detect parkinsonian syndromes and monitor disease progression (Cochrane and Ebmeier, 2013). The novel contribution of this study is that an acute dose of dopaminergic medication failed to alter the free-water and FA_t_ measures from the basal ganglia regions. In a longitudinal study, free-water in the posterior substantia nigra increased over one year in PD, but not in a control group (Ofori et al., 2015b). When using a single tensor diffusion tensor model, progression effects have also been observed in the substantia nigra (Loane et al., 2016). In a prior study examining a retrospective cohort of PD treated with rasagiline compared with PD not treated with rasagiline (Burciu et al., 2016), it was found that PD patients who had not taken rasagiline had significantly higher free-water in the posterior substantia nigra than PD patients who had taken rasagiline and healthy controls. Further, PD patients who had taken rasagiline had significantly higher free-water in the posterior substantia nigra than healthy controls (Burciu et al., 2016). The main differences between the prior study and the current one, is that patients were chronically taking rasagiline, whereas the current study examined a single dose of carbidopa/levodopa and other medications. Collectively, these results suggest that dMRI measurements in the basal ganglia most likely reflect the chronic state of microstructural integrity and do not seem to be altered by short-term, dopaminergic medication.

There were some caveats in the present study. The current study focused on the effects of acute dopaminergic medication on changes of dMRI. It is possible that longer-term use of dopaminergic medication could influence dMRI measurements. An example of this experimental paradigm from studies in rodents found that 4 weeks of dopaminergic treatment affected ^11^C-dihydrotetrabenazine (DTBZ, VMAT2 marker) and ^11^C-methylphenidate (MP, DAT marker) (Sossi et al., 2010). It is also the case that DTBZ, VMAT2, MP, and DAT are functional biomarkers from positron emission tomography, whereas the current biomarker is structural in nature and does not use a contrast agent. In addition, although we found significant MDS-UPDRS-III changes between ON and OFF-medication conditions, the mean changes were on the order of a few points because the patients were mild, and it is possible that moderate stage patients with a stronger drug response could lead to a different result. Studies with drug-naïve subjects and controlled doses of medication, as well as patients in various stages of the PD disease are needed to confirm the null changes of dMRI by dopaminergic medication.

In summary, we have shown for the first time that free-water and FA_t_ in the basal ganglia are not influenced by administration of acute antiparkinsonian medication. These findings provide evidence that free-water, a proposed progression marker (Ofori et al., 2015b) is not affected by a patient-specific acute dose of antiparkinsonian medication, suggesting that this marker is measuring the chronic state of the basal ganglia. Collectively, these neuroimaging measurements such as dMRI free-water and FA_t_, are well-suited disease progression biomarkers to reflect the chronic state of basal ganglia in PD. These biomarkers could be useful in clinical trials testing disease-modifying therapies for PD.
